# The Sense of Smell and Aging: What We Have Learned From Population Studies

**DOI:** 10.1093/geroni/igaa057.2947

**Published:** 2020-12-16

**Authors:** Jayant Pinto

**Affiliations:** University of Chicago, Chicago, Illinois, United States

## Abstract

Decline of the sense of smell with age causes a marked impact on older adults, markedly reducing quality of life. Olfactory dysfunction impairs nutrition, decreases the ability to experience pleasure, and results in depression, among other burdens. Large-scale population studies have identified impaired olfaction as a key heath indicator that predicts the development of decreased physical and mental health, reduced physical activity, weight loss, mild cognitive impairment and dementia, and mortality itself. These data have been generated via analyses of data from several aging cohorts, including the National Social Life, Health, and Aging Project (NSHAP); the Beaver Dam cohort; the Atherosclerosis Risk in Communities project; the Rush Memory and Aging Project; the Health, Aging, and Body Composition project; the Washington Heights/Inwood Columbia Aging Project; among others. In this presentation, we will review the close connection between olfaction, health, aging, including discussion of insights from these studies. We will also discuss emerging data from NSHAP on the effects of sensory function on cognition, mental health, and social interaction, which demonstrate that sensory function plays a vital role in the lives of older adults. Part of a symposium sponsored by Sensory Health Interest Group.

